# Experimental Models of Type 2 Diabetes Mellitus Induced by Combining Hyperlipidemic Diet (HFD) and Streptozotocin Administration in Rats: An Integrative Review

**DOI:** 10.3390/biomedicines13051158

**Published:** 2025-05-09

**Authors:** Ana Karolinne da Silva Brito, Ana Victória da Silva Mendes, Boris Timah Acha, Amanda Suellenn da Silva Santos Oliveira, Joyce Lopes Macedo, Akemi Suzuki Cruzio, Maria das Graças Prianti, Raquel Rodrigues de Abreu, Massimo Lucarini, Alessandra Durazzo, Maria do Carmo de Carvalho e Martins, Daniel Dias Rufino Arcanjo

**Affiliations:** 1 LAFMOL—Laboratory of Functional and Molecular Studies in Physiopharmacology, Department of Biophysics and Physiology, Federal University of Piauí, Teresina 64049-550, PI, Brazil; karolinnebrito@ufpi.edu.br (A.K.d.S.B.); victoriams18@hotmail.com (A.V.d.S.M.); timah.boris@yahoo.com (B.T.A.); amandasuellenn@hotmail.com (A.S.d.S.S.O.); joycelopes385@gmail.com (J.L.M.); carminhamartins@ufpi.edu.br (M.d.C.d.C.e.M.); 2 Faculty of Pharmacy, CET—College of Technology of Teresina, Teresina 64003-420, PI, Brazil; akemiscruzio@gmail.com (A.S.C.); mgprianti@gmail.com (M.d.G.P.); raquelramon01@gmail.com (R.R.d.A.); 3 CREA—Research Centre for Food and Nutrition, Via Ardeatina 546, 00178 Rome, Italy; massimo.lucarini@crea.gov.it

**Keywords:** animal model, type 2 diabetes mellitus, high-fat diet, streptozotocin

## Abstract

Type 2 diabetes mellitus (DM2) is a metabolic disorder characterized by chronic hyperglycemia associated with low insulin production and/or insulin resistance. A high-fat diet (HFD) combined with a low dose of streptozotocin (STZ) in an animal model produces a disease that mimics type 2 diabetes mellitus in humans. However, there is wide variation in the methods of inducing diabetes in terms of the dose of STZ, the duration of the induction period, and the composition of the diet used, all of which could result in biological responses that are not typical of the disease. This review aims to investigate the characteristics of an experimental model of type 2 diabetes mellitus by combining a high-fat diet with low doses of streptozotocin in Wistar rats. This is an integrative review conducted by searching in the Medline, Lilacs, and Embase databases using the keywords “type 2 diabetes mellitus”, “high-fat diet”, “streptozotocin” and “Wistar rats”. Articles published in English between 2018 and 2025 were included. The induction of DM2 in young male rats with a high-fat HFD for a period of at least 3 weeks followed by a low dose of STZ resulted in metabolic, histological, inflammatory, and oxidative changes, and alterations in the signaling pathways of glycemic and lipid metabolism in different tissues, replicating the characteristics observed in humans. HFD-fed + STZ-induced Wistar rats constitute an effective animal model for studying DM2.

## 1. Introduction

Diabetes mellitus is a disease characterized by chronic hyperglycemia due to insulin deficiency and/or resistance to the action of this hormone, and its progression is associated with the occurrence of macro- and microvascular complications in various organ systems. Type 2 diabetes mellitus is the most common form of this disease and, although its etiology is multifactorial, lifestyle factors such as sedentary lifestyle and poor diet appear to be strongly associated with its development [1].

According to estimates, one in 10 adults suffer from this disease worldwide, corresponding to 537 million people. This number is expected to increase to 643 million by 2030 and 783 million by 2045. Additionally, diabetes and its complications are among the leading causes of mortality worldwide, with an estimated 6.7 million deaths in people aged 20–79 years in 2021, and a third of those deaths occurring in those under 60 [2].

The problems surrounding this disease have led to numerous studies that are being carried out with the aim of producing and identifying the mechanisms of action of new therapeutic tools. The use of animal models plays an important role in the production of knowledge, enabling the study of target organs and tissues, which contributes to obtaining relevant data in less time and at lower costs, as well as generating results that support clinical trials and translational applications [3,4].

In order for an experimental model of diabetes mellitus to be considered appropriate, it must exhibit pathophysiological alterations and result in the development of complications similar to those found in humans. Several animal models have been described for type 2 diabetes mellitus, and there is no single model that includes all of its pathological and etiological characteristics. In this sense, key aspects in characterizing this model for pathophysiological studies are the presence of insulin resistance associated with the reduced secretion of this hormone by the beta cells of the pancreatic islets [3,5].

These characteristics are based on the twin cycle hypothesis, in which the high energy consumption associated with insulin resistance leads to an increase in plasma triglycerides and hepatic steatosis. In this cycle, the excess fat in the liver hinders hepatic responsiveness to insulin, reducing glycogen synthesis and increasing gluconeogenesis, leading to hyperglycemia. Conversely, hypertriglyceridemia produces lipotoxicity in the pancreatic islets and a consequent reduction in insulin secretion. Together, these processes exacerbate hyperglycemia and lead to pancreatic beta-cell failure [6].

The induction of type 2 diabetes mellitus using a high-fat diet (HFD) combined with a low dose of streptozotocin (STZ) (2-deoxy-2-(3-methyl-3-nitrosourea)-1-D-glucopyranose), a beta cytotoxin, aims to develop a rat model with the characteristics of type 2 diabetes in a short period of time. A high-fat diet will produce insulin resistance because of excess weight and obesity, while the low dose streptozotocin is designed to cause damage to part of the pancreatic beta cells. An animal develops diabetes due to the combination of insulin resistance and the reduced production of this hormone [5].

Among the inducible experimental models described in the literature, this model of type 2 diabetes is considered to be the one that best mimics the characteristics of the disease. Chao et al. [7], by comparing four type 2 diabetes induction models, observed that although higher HOMA-IR (Homeostasis Model Assessment of Insulin Resistance) and plasma insulin values were observed in the fructose induction model, this model did not produce significant changes in blood glucose. In contrast, the HFD + STZ model produced the highest blood glucose values and the second highest change in HOMA-IR, as well as insulin resistance in the hyperinsulinemic–euglycemic clamp test and a significant reduction in the expression of the GLUT4 glucose transporter [7].

On the other hand, a variety of ways of inducing type 2 diabetes using the HFD + STZ model have been described by different authors, with variations in the doses of STZ, induction times, and diet composition [8,9,10]. These variations produce pathological characteristics that can range from mild, with glucose intolerance, to severe, with severe glycemic decompensation [11]. Based on the assumption that type 2 diabetes can be mischaracterized depending on the type of alteration identified in glycemic metabolism, this study aimed at characterizing the experimentally induced models of type 2 diabetes mellitus using a high-fat diet and low-dose streptozotocin administration in Wistar rats, which exhibit characteristics similar to the clinical manifestations of this disease in humans. The integrative review was selected to respond to this objective given its role in providing evidence that allows for a comprehensive understanding of the subject under study and the inclusion of different experimental designs [12].

## 2. Materials and Methods

Our investigation is an integrative literature review based on this guiding question: “What are the characteristics of models for inducing type 2 diabetes mellitus in Wistar rats using an HFD combined with SZT to develop a model more similar to that observed in humans?”. Studies were located/identified by searching the Medline, Embase, and Lilacs databases using the following keywords: “type 2 diabetes mellitus”, “high-fat diet”, “streptozotocin”, and “wistar rats”, adopting the following as the search strategy: (type 2 diabetes mellitus) AND (streptozotocin) AND (high fat diet) AND (wistar rat).

Original articles published in English between January 2018 and February 2025 that reported on the induction of type 2 diabetes mellitus in Wistar rats using a high-fat diet and low-dose streptozotocin administration were included. Articles were excluded if the macronutrient content or duration of the diet, as well as the streptozotocin dosage, were not mentioned; if nutrients except fat were added to the diet; if another induction model that also uses HFD or STZ or if other pathologies associated with diabetes were induced; if the results did not compare normal and diabetic subjects; or if insulin resistance or glucose tolerance markers were not measured.

Articles were selected by reading the titles and abstracts for initial screening, and then reading the full text of those considered eligible. All original studies that used the STZ + HFD model to test for any antidiabetic treatment and that presented the characteristics defined in the inclusion criteria were included in the results. Only comparisons between normal and diabetic groups without treatment were analyzed. Articles that met the established criteria and aimed to standardize a new induction model by testing different doses of STZ and/or diet compositions or induction periods were included in the discussion of this study only when necessary.

The SYRCLE tool was used to assess the risk of bias regarding the included experimental studies [13]. This tool consists of ten questions divided into the following evaluation categories: selection bias, performance bias, detection bias, attrition bias, reporting bias, and other sources of bias. The answers obtained were classified as “YES”, which indicates a low risk of bias; “NO”, which indicates a high risk of bias; and “UNCERTAIN”, which indicates insufficient details to adequately assess the risk of bias.

## 3. Results

**Figure 1:**
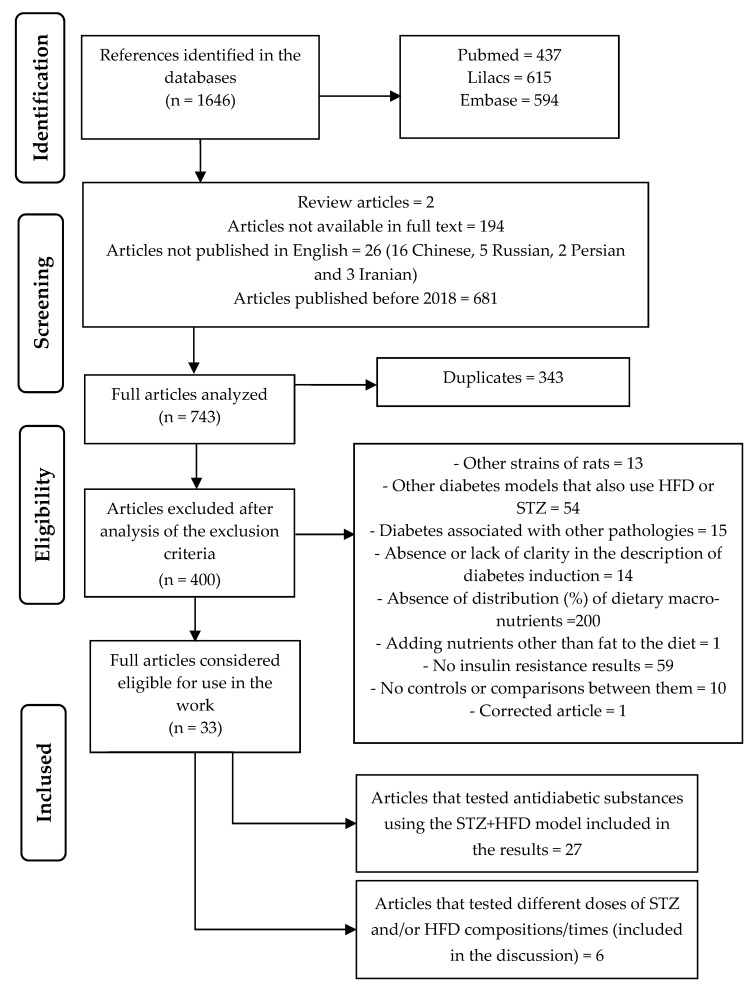
Flow Diagram of Study Identification and Screening Workflow.

A total of 1646 articles was obtained from the search in the different databases. After the selection and removal of articles that did not meet the inclusion criteria, 29 studies were considered eligible to compose the results of this study. Figure 1 shows a flowchart detailing the selection of articles and the corresponding search results.

Table 1 shows the articles included in the results of this review by author, year and country of publication, the method of diabetes induction, and the results obtained. Most of the studies included male rats between 4 and 24 weeks old, weighing between 150 and 280 g.

The initial supply of the high-fat diet varied from 14 to 91 days, with the 28-day period being the most frequent, as reported in 29.6% of the studies. Depending on the diet’s composition, the percentage of carbohydrates ranged from 17 to 48%, proteins from 10 to 25%, and lipids from 22 to 60% of the total weight of the diet. The sources of fat in the diet were mostly saturated fatty acids, including lard and soybean oil [14,15], lard [16,17], butter [18,19], sheep butter [20], corn oil and beef tallow [21], lard oil [22], rump oil [23], and lard and cholesterol [24]. Streptozotocin was administered intraperitoneally as a single dose, at doses ranging from 25 to 45 mg/kg; 35 mg/kg was the most commonly used dose (as reported in 55.5% of the studies).

The most frequent criteria for confirming the diagnosis of diabetes described in the studies were fasting blood glucose measurements, using blood glucose levels above the 140 to 300 mg/dL range as the cut-off point for diagnosing DM. Two of the studies used random blood glucose measurements with cutoffs above the 300 mg/dL range, and two studies used an oral glucose tolerance test, one of them assuming that animals with blood glucose above 144 mg/dL after 120 min of glucose overload were diabetic, while the other did not specify the cutoff point. Seven of the studies using the 126 to 300 mg/dL range did not report the type of blood glucose used. Periods for blood glucose monitoring to confirm diabetes ranged from 3 to 28 days after streptozotocin administration, and the follow-up of animals after confirmation of diabetes ranged from 7 to 140 days.

The outcome assessed in the selected studies include analysis of body weight and food and water consumption; diuresis; glycemic control; lipid profile; liver, kidney and heart function; markers of inflammation, oxidative damage and antioxidant activity; hormonal function; the histopathology of the liver, pancreas and adipose tissue; gene and protein expression related to insulin signaling pathways, inflammatory pathways, and intermediary glucose metabolism pathways (glycogenolysis and gluconeogenesis); lipid metabolism (fatty acid synthesis); and the dysregulation of pathway transcriptional control, apoptosis, and cell proliferation.

Table 2 shows the results of the methodological quality (risk of bias) assessment of this study. None of the studies clearly described the sequence to ensure randomization and blinding in animal distribution, housing, and intervention (questions 1, 3, 4, and 5). Only three studies provided complete information on the baseline characteristics of the animals, including the glycemia of the animals before DM2 induction (question 2); two studies adequately described the selection of animals used (question 6); and one study specified that the evaluator was blinded to the analysis of the results (question 7).

More than half of the studies adequately addressed the results, showing coherence between the methods and results regarding the number of animals used (question 8), and most of the studies reported all of their results, showing consistency between the methods and the results (question 9). Regarding other types of bias, 74% included clearly defined cut-off points for confirming diabetes (question 10), 33.3% clearly reported the results obtained in confirming diabetes (question 11), and 88.8% clearly described the glycemic results of the intervention period (question 12).

**Table 1:** Studies on the induction of type 2 diabetes in Wistar rats using streptozotocin and a high-fat diet by author, study methodology, and observed outcomes.

Reference	Methodology Induction Protocol	Outcomes *
Bem et al. [14]	- Male Wistar rats weighing between 180 and 200 g. - Consumption of HFD (carbohydrate: 31%; protein: 14%; fat: 55%; lipid source: lard and soybean oil) for 21 days. - Administration of STZ (35 mg/kg i.p., single dose). - Confirmation of DM by glycemia (not disclosed) 14 days after STZ: DM if glycemia > 210 mg/dL. - Intervention for 28 days.	METABOLIC CONTROL: ↔ body weight after confirmation of DM and at the end of the intervention. GLYCEMIC CONTROL: ↑ blood glucose after confirmation and at the end of the intervention; ↑ HbA1c; ↑ insulin; ↑ HOMA-IR, ↓ HOMA-β. LIPID PROFILE: ↑ TG, ↑ TC, ↑ VLDL, ↔ LDL-c, ↓ HDL-c. INFLAMMATORY MARKERS: ↑ IL-6, ↑ TNF-α HORMONAL FUNCTION: ↑ Leptin, no change in GLP1 PROTEIN EXPRESSION: - Protein expression in soleus muscle: ↓ IR, ↔ AKT, ↓ p-AKT, ↓ GLUT4, ↓ p-AMPK. - Protein expression in visceral adipose tissue: ↑ IR, ↔ AKT, ↓ pAKT, ↓ GLUT4, ↓ adiponectin. VASCULAR REACTIVITY: ↓ Acetylcholine-induced vasodilation, ↔ nitroglycerin-induced vasodilation, ↑ norepinephrine-induced vasoconstriction in the mesenteric artery.
Gheibi et al. [18]	- Male Wistar rats weighing between 190 and 210 g. - Consumption of HFD (carbohydrate: 27.5%; protein: 14.7%; fat: 58.8%; lipid source: butter) for 14 days. - Administration of STZ (25 mg/kg body weight, i.p., single dose). - Confirmation of DM by fasting glucose levels 7 days after STZ: DM if glucose level ≥ 150 mg/dL. - Intervention for 49 days.	METABOLIC CONTROL: ↑ body weight, ↑ calorie intake, ↑ water intake, ↓ food consumption. GLYCEMIC CONTROL: ↑ Serum glucose, ↑ serum insulin, ↑ HbA1c; ↑ area under the curve of serum glucose and serum insulin in the intraperitoneal (i.p.) glucose tolerance test; ↑ area under the curve of serum glucose in the i.p. pyruvate tolerance test; ↑ area under the curve of serum glucose in the i.p. insulin tolerance test. LIPID PROFILE: ↑ TG, ↑ TC, ↑ LDL-c, ↑ HDL-c. INFLAMMATORY MARKERS: ↑ serum IL-1β OXIDATIVE AND ANTIOXIDANT MARKERS: ↑ serum MDA; ↓ CAT; ↔ nitrite, nitrate and serum NOx. HISTOPATHOLOGICAL: ↓ content and ↓ secretion of insulin in the pancreatic islets. GENE AND PROTEIN EXPRESSION: - ↑ iNOS mRNA expression, ↓ GLUT4 mRNA expression, and ↓ GLUT4 protein expression in the soleus muscle; - ↑ iNOS mRNA expression, ↓ GLUT4 mRNA expression, and ↓ GLUT4 protein expression in epididymal adipose tissue.
Sathiyabama et al. [25]	- Male Wistar rats weighing between 180 and 200 g. - Consumption of HFD (carbohydrate: 31%; protein: 14%; fat: 55%; lipid source: not disclosed) for 14 days. - Administration of STZ (40 mg/kg i.p., single dose). - Confirmation of DM by fasting glucose levels 5 days after STZ: DM if glucose levels > 250 mg/dL. - Intervention for 30 days.	METABOLIC CONTROL: ↑ weight over the 30 days of intervention; ↑ food consumption, ↑ water intake. GLYCEMIC CONTROL: ↑ glycemia over the 30 days of intervention; ↑ serum insulin, ↑ HOMA-IR, ↑ glycemia after 120 min in the oral glucose tolerance test, ↑ glycemia after 60 min in the i.p. insulin tolerance test. LIPID PROFILE: ↑ TG, ↑ TC, ↑ LDL-c, ↓ HDL-c. HEPATIC FUNCTION: ↑ ALT, ↑ AST, ↑ Alkaline phosphatase. RENAL FUNCTION: ↑ urea, ↑ creatinine. HISTOPATHOLOGICAL: Pancreatic β-cells with generalized degranulation and vacuolization, and disordered islet architecture; ↓ insulin content in the pancreatic islet; enalarged epididymal adipose tissue cells. GENE AND PROTEIN EXPRESSION: - mRNA expression in adipose tissue: ↓ PPARγ, ↓ GLUT4, ↓ IR. - Protein expression in adipose tissue: ↓ PPARγ, ↓ GLUT4 and ↓ IR.
Sohrabipour et al. [26]	- Male Wistar rats aged 4 weeks. - Consumption of HFD (carbohydrate: 17%; protein: 25%; fat: 58%; lipid source: (not disclosed) for 28 days. - Administration of STZ (35 mg/kg i.p., single dose). - Confirmation of DM by fasting glucose levels 3 days after STZ: DM if glucose levels > 250 mg/dL in 3 consecutive measurements. - Intervention for 90 days.	METABOLIC CONTROL: ↓ body weight at the end of the intervention; ↓ abdominal fat; ↓ food consumption, ↑ water intake, and ↑ urinary excretion at 30, 60, and 90 days of intervention. GLYCEMIC CONTROL: ↑ random blood glucose over 90 days of intervention; ↑ area under the serum glucose curve in the i.p. glucose tolerance test after 30 and 90 days of intervention; ↑ area under the serum glucose curve in the i.p. insulin tolerance test after 30 and 90 days of intervention; ↓ glucose infusion rate necessary to maintain normoglycemia during insulin infusion in the euglycemic–hypersulinemic clamp; ↓ plasma insulin, ↑ plasma glucagon. GENE EXPRESSION: - mRNA expression in the gastrocnemius muscle and liver: ↑ glucagon receptor; ↑ PEPCK; ↑ G6pase; ↔ GLUT4.
Abel-Hamid et al. [21]	- Male Wistar rats weighing 150–180 g and aged 6–8 weeks. - Consumption of HFD (carbohydrate: 24%; protein: 20%; fat: 46%; lipid source: corn oil and beef tallow) for 28 days. - Administration of STZ (35 mg/kg i.p., single dose). - Confirmation of DM by glycemia (not disclosed) 7 days after STZ: DM if glycemia > 200 mg/dL. - Intervention for 7 days.	METABOLIC CONTROL: ↑ body weight, ↑ food consumption, ↑ water intake, ↑ visceral fat weight. GLYCEMIC CONTROL: ↑ fasting glucose, ↓ serum insulin, ↑ HOMA-IR. MARKERS OF INFLAMMATION: ↑ serum IL-6. HISTOPATHOLOGICAL: ↓ hepatic glycogen content. GENE EXPRESSION: - mRNA expression in the hypothalamus: ↑ NPY. - mRNA expression in visceral fat: ↑ NF-kB.
Ghiasi et al. [27]	- Male Wistar rats weighing between 200 and 250 g. - Consumption of HFD (carbohydrate: 48%; protein: 20%; fat: 22% (does not amount to 100%; source of lipid: not disclosed) for 28 days. - Administration of STZ (35 mg/kg i.p., single dose). - Confirmation of DM by random glucose levels 7 days after STZ: DM if glycemia > 300 mg/dL. - Intervention for 84 days.	METABOLIC CONTROL: ↓ body weight. GLICEMIC CONTROL: ↑ fasting glucose, ↑ serum insulin, ↓ QUICKI. LIPID PROFILE: ↑ TG, ↑ TC, ↓ HDL-c. HEPATIC FUNCTION: ↓ serum albumin. OXIDATIVE AND ANTIOXIDANT MARKERS: ↑ MDA, ↓ SOD, ↓ Gpx, ↓ pancreatic CAT. HISTOPATHOLOGICAL: ↓ pancreatic beta-cell density. GENE AND PROTEIN EXPRESSION mRNA expression in the pancreas: ↓ SIRT1. Protein expression in the pancreas: ↓ SIRT1.
Omidi et al. [28]	- Male Wistar rats weighing > 250 g and aged 20–24 weeks. - Consumption of HFD (carbohydrate: 48%; protein: 20%; fat: 32%; lipid source: not disclosed) for 30 days. - Administration of STZ (35 mg/kg i.p., single dose). - Confirmation of DM by glycemia (not disclosed) 7 days after STZ: DM if glycemia > 250 mg/dL. - Intervention for 56 days.	GLYCEMIC CONTROL: ↑ fasting glucose, ↑ serum insulin, ↑ HOMA-IR. LIPID PROFILE: ↔ TG, TC, LDL-c, and HDL-c.
Xiang et al. [22]	- Male Wistar rats weighing between 200 and 220 g. - Consumption of HFD (carbohydrate: 35%; protein: 20%; fat: 45%; lipid source: lard oil) for 28 days. - Administration of STZ (30 mg/kg i.p., single dose). - Confirmation of DM by fasting glycemia 28 days after STZ: DM if glucose levels > 140 mg/dL. - Intervention for 30 days.	METABOLIC CONTROL: ↓ body weight, ↓ weight gain; ↔ food consumption, ↓ food utilization rate and ↑ water intake at the end of the intervention; ↑ liver weight, ↔ epididymal fat weight. GLYCEMIC CONTROL: ↑ serum glucose, ↑ HbA1c, ↑ serum insulin, ↑ HOMA-IR, ↓ QUICKI; ↑ area under the serum glucose curve in the i.p. glucose tolerance test, **↑** area under the serum glucose curve in the i.p. insulin tolerance test. LIPID PROFILE: ↑ TG, ↔ TC, LDL-c and non-esterified fatty acids (NEFA), ↓ HDL-c. LIVER FUNCTION: ↑ ALT. METABOLOMIC ANALYSIS: metabolic disorder evidenced by clear separation of hepatic metabolites found in normal and diabetic controls. GENE AND PROTEIN EXPRESSION - mRNA expression in the liver: ↓ Irs1, ↓ aAKT2, ↓ Mtor, ↓ Rptor. - Relative protein expression ratio in the liver: ↓ p-AKT/AKT, ↓ p-FOXO1/FOXO1. - Protein expression in the liver: ↑ SREBP1, ↑ FAS, ↑ PCK2 and ↑ FOXO1.
Zhang et al. [29]	- Male Wistar rats weighing between 180 and 200 g. - Consumption of HFD (carbohydrate: 20%; protein: 20%; fat: 60%; lipid source: not disclosed) for 84 days. - Administration of STZ (28 mg/kg i.p., single dose). - Confirmation of DM by fasting glycemia 3 days after STZ: DM if glucose levels > 200 mg/dL. - Intervention for 35 days.	METABOLIC CONTROL: ↓ body weight over the 35 days of intervention. GLYCEMIC CONTROL: ↑ fasting blood glucose, ↑ fasting serum insulin, ↑ HOMA-IR, ↓ HOMA-IS; ↑ area under the serum glucose curve in the oral glucose tolerance test. LIPID PROFILE: ↑ TG, ↑ CT. HORMONAL FUNCTION: ↑ Leptin. MARKERS OF INFLAMMATION: ↓ Adiponectin. HISTOPATHOLOGICAL: Steatotic liver with vacuolization accompanied by lobular inflammation and balloon-like changes in the hepatocytes; ↑ hepatic TG. GENE AND PROTEIN EXPRESSION: - mRNA expression in liver: ↓ PPARγ, ↑ DGAT2. - Protein expression in liver: ↓ PPARγ, ↑ DGAT2.
Lv et al. [30]	- Male Wistar rats weighing between 180 and 220 g. - Consumption of HFD (carbohydrate: 35%; protein: 20%; fat: 45%; lipid source: not disclosed) for 28 days. - Administration of STZ (30 mg/kg i.p., single dose). - Confirmation of DM by fasting glucose 28 days after STZ (cut-off point for diabetes not determined). - Intervention for 28 days.	METABOLIC CONTROL: ↓ weight after STZ application, ↑ food consumption, ↑ water intake. GLYCEMIC CONTROL: ↑ serum glucose, ↑ HbA1c, ↑ serum insulin, ↑ HOMA-IR; ↑ the area under the serum glucose curve in the i.p. glucose tolerance test, **↑** the area under the serum glucose curve in the i.p. insulin tolerance test. LIPID PROFILE: ↑ TG, ↑ TC, ↔ HDL-c, LDL-c, and non-esterified fatty acids (NEFAs). MARKERS OF INFLAMMATION: ↑ IL-1β, ↑ IL-6, ↑ IL-8, ↑ TNF-α. HISTOPATHOLOGICAL: Altered architecture of the hepatic lobules; hepatocytes increased their sizes and exhibited blurred borders. Cytoplasm filled with fat vacuoles of different sizes, pushing the nucleus to the periphery. TRANSCRIPTOMA ANALYSIS: Trend of spatial separation in the analysis of RNA sequences in the livers of normal controls and diabetics, suggesting a significant difference in gene expression.
Mangali et al. [31]	- Male Wistar rats weighing 180–220 g and aged 5–6 weeks. - Consumption of HFD (carbohydrate: 17%; protein: 25%; fat: 58%; lipid source: (not disclosed) for 28 days. - Intraperitoneal glucose tolerance test and intraperitoneal insulin tolerance test. - Administration of STZ (35 mg/kg i.p., single dose) in rats with insulin resistance. - Confirmation of DM by fasting glycemia 3 days after STZ: DM if glycemia ≥ 250 mg/dL. - Intervention for 56 days.	METABOLIC CONTROL: ↔ body weight. GLYCEMIC CONTROL: ↑ blood glucose; ↑ serum glucose after 120 min in the i.p. glucose tolerance test; ↑ serum glucose after 120 min in the i.p. insulin tolerance test. CARDIOVASCULAR FUNCTION: ↑ Creatine kinase-MB (CK-MB); ↑ lactate dehydrogenase (LDH); ↑ heart rate; ↑ systolic pressure; ↑ diastolic pressure; ↑ heart weight; ↑ relative heart weight; ↑ cardiac fibrosis area. LIPID PROFILE: ↑ TG, ↑ TC, ↑ LDL-c, ↓ HDL-c. MARKERS OF INFLAMMATION: ↑ TNF-α, ↑ NF-kB, ↑ c-Jun-N-terminal kinase (JNK) in cardiac muscle OXIDATIVE AND ANTIOXIDANT MARKERS: ↑ generation of reactive oxygen species (DCFDA test); ↑ advanced glycation end products (AGEs) in cardiac muscle. PROTEIN EXPRESSION: Protein expression in cardiac muscle: ↑ PKR, ↑ α-SMA; ↑ TGF-β; ↑ Casp-3; ↑ ERK1/2 and ↑ p-ERK1/2.
Xu et al. [32]	- Male Wistar rats weighing between 180 and 220 g. - Consumption of HFD (carbohydrate: 41%; protein: 24%; fat: 24%; lipid source: not disclosed) for 28 days. - Administration of STZ (30 mg/kg i.p., single dose). - Confirmation of DM by random glucose levels 3 days after STZ: DM if glycemia > 300 mg/dL. - Intervention for 56 days.	METABOLIC CONTROL: ↓ body weight over the 56 days of intervention; ↑ food consumption, ↑ water intake. GLYCEMIC CONTROL: ↑ fasting glucose; ↑ insulin; ↑ HOMA-IR; ↓ insulin sensitivity index (ISI); ↑ HbA1c; ↓ hepatic glycogen; ↑ area under the serum glucose curve in the oral glucose tolerance test; ↑ % of serum glucose in the i.p. insulin tolerance test after 30, 60, and 90 min. LIPID PROFILE: ↑ TG, ↑ TC, ↑ LDL-c, ↓ HDL-c, ↑ free fatty acids (FFAs). HEPATIC FUNCTION: ↑ ALT, ↑ AST, ↑ AST/ALT ratio. GENE AND PROTEIN EXPRESSION - ↓ relative miR-125a-5p expression; ↑ signal transducer and activator of transcription 3 (STAT3) mRNA in the liver. - Protein expression in the liver: ↑ STAT3; ↑ p-STAT3; ↑ PEPCK; ↑ G6pase; ↑ SREBP1c; ↑ FAS; ↑ ACC; ↑ SCD1. - Relative protein expression ratio in the liver: ↓ p-PI3k/PI3K; ↓ p-AKT/AKT; ↓ p-GSK3β/GSK3β; ↓ p-FOXO1/FOXO1.
Kolefer; Miaffo; Ponka, [33]	- Male Wistar rats weighing between 200 and 250 g and aged 16–17 weeks. - Consumption of HFD (carbohydrate: 17%; protein: 25%; fat: 58%; lipid source: not disclosed) for 30 days. - Body mass index (BMI) analysis. - Administration of STZ (35 mg/kg i.p., single dose) in rats with BMI > 0.7 g/cm^2^. - Confirmation of DM by glycemia (not disclosed) 3 days after STZ: DM if glycemia > 126 mg/dL. - Intervention for 28 days.	METABOLIC CONTROL: ↑ body weight; ↑ BMI; ↑ abdominal fat. GLYCEMIC CONTROL: ↑ glycemia over 28 days of intervention; ↑ insulin, ↑ HOMA-IR, ↓ HOMA-B. LIPID PROFILE: ↑ TG, ↑ TC, ↑ VLDL, ↑ LDL-c, ↓ HDL-c; ↑ atherogenic index (Log[TG/HDL-c]); ↑ coronary heart risk index (CT/HDL-c); ↓ cardioprotective index (LDL-c/HDL-c). LIVER FUNCTION: ↑ ALT, ↑ AST. RENAL FUNCTION: ↑ urea, ↑ creatinine. OXIDATIVE AND ANTIOXIDANT MARKERS: ↑ MDA, ↓ SOD, ↓ GSH, ↓ CAT in liver, heart, and kidney tissues.
Rezazadeh et al. [24]	- Male and female Wistar rats aged 4 weeks - Consumption of HFD (carbohydrate: 17%; protein: 25%; fat: 58%; lipid source: lard and cholesterol) for 84 days. - Administration of STZ (35 mg/kg i.p., single dose). - Confirmation of DM by glycemia (not disclosed) 7 days after STZ: DM if glycemia ≥ 250 mg/dL. - Intervention for 140 days.	METABOLIC CONTROL: ↓ body weight at the end of the intervention; ↑ water intake and ↑ diuresis at 28 days, 56 days, and 84 days after the intervention. GLYCEMIC CONTROL: ↑ glycemia after confirmation of diabetes and over 140 days of intervention. - ↑ HbA1c at the end of the intervention; ↑ area under the serum glucose curve in the i.p. glucose tolerance test at 28 days, 56 days, and 84 days after the intervention; ↓ glucose infusion rate required to maintain normoglycemia during insulin infusion in the euglycemic–hypersulinemic clamp at the end of the intervention. GENE EXPRESSION: - mRNA expression in gastrokinemius muscle: ↓ IRS1, ↓ AKT, ↓ GLUT4.
Zelinskaya et al. [34]	- Male Wistar rats aged 8 weeks - Consumption of HFD (carbohydrate: 38%; protein: 18%; fat: 22%; lipid source: not disclosed) for 20 days. - Administration of STZ (20 mg/kg body weight, i.p., single dose). - Oral glucose tolerance test (2 g/kg) after 14 days (diabetic rats if blood glucose levels ≥ 144 mg/dL after 120 min). - Intervention for 49 days.	GLYCEMIC CONTROL: ↑ area under the serum glucose curve in the oral glucose tolerance test. VASCULAR REACTIVITY - ↔ % of phenylephrine-induced contraction in the femoral and mesenteric arteries; - ↔ % of serotonin-induced contraction in the femoral and mesenteric arteries; - The presence of protoporphyrin (a guanylyl cyclase inhibitor) abolishes the effect of acetylcholine in the mesenteric artery, but not in the femoral artery.
Khoramipour et al. [15]	- Male Wistar rats weighing between 189, 75 and 210, 25 g and aged 8 weeks. - Consumption of HFD (carbohydrate: 20%; protein: 19%; fat: 60%; lipid source: lard and soybean oil) for 56 days. - Administration of STZ (35 mg/kg i.p., single dose). - Confirmation of DM by fasting glycemia 3 days after STZ: DM if glycemia > 300 mg/dL. - Intervention for 56 days.	METABOLIC CONTROL: ↑ body weight after the high-fat diet and STZ injection, ↔ body weight after the 56 days of intervention, ↓ food consumption in grams and calories over the 56 days of intervention, ↑ water intake over the 56 days of intervention. GLYCEMIC CONTROL: ↑ fasting glucose after the high-fat diet and STZ injection, ↑ fasting glucose after the 56 days of intervention, ↓ insulin; ↑ HOMA-IR. HORMONAL FUNCTION: ↓ Leptin. PROTEIN EXPRESSION: Protein expression in the hypothalamus: ↓ LEP-R, ↑ JAK-2, ↓ p-JAK-2, ↑ STAT3, ↓ p-STAT3, ↓ POMC, ↓ CART, ↑ NPY, ↑ AGRP, ↑ FOXO1, ↑ SOCS3.
Vijay; Vellapandian [16]	- Male Wistar rats weighing between 150 and 200 g. - Consumption of HFD (carbohydrate: 17%; protein: 25%; fat: 58%; lipid source: lard) for 21 days. - Administration of STZ (35 mg/kg i.p., single dose). - Confirmation of DM by fasting glycemia 3 days after STZ: DM if glycemia > 200 mg/dL. - Intervention for 21 days.	METABOLIC CONTROL: ↑ body weight over the 21 days of intervention. GLYCEMIC CONTROL: ↑ glycemia over the 21 days of intervention, ↓ insulin; ↑ HOMA-IR; ↑ area under the serum glucose curve in the oral glucose tolerance test. LIPID PROFILE: ↑ TG, ↑ TC, ↑ LDL-c, ↓ HDL-c. LIVER FUNCTION: ↑ ALT, ↑ AST, ↑ ALP. RENAL FUNCTION: ↑ urea, ↑ creatinine. OXIDATIVE AND ANTIOXIDANT MARKERS: ↑ MDA, ↓ SOD, ↓ GSH, ↓ CAT in gastrocnemius muscle. HISTOPATHOLOGICAL: - Pancreatic tissue—degeneration of pancreatic islets with reduced cell size and number and loss of pancreatic architecture. - Hepatic tissue—moderate congestion and accumulation of lipids.
Salem et al. [17]	- Male Wistar rats weighing between 150 and 180 g - Consumption of HFD (carbohydrate: 17%; protein: 25%; fat: 58%; lipid source: lard) for 14 days. - Administration of STZ (35 mg/kg i.p., single dose). - Confirmation of DM by fasting glycemia 3 days after STZ: DM if glycemia > 150 mg/dL. - Intervention for 42 days.	GLYCEMIC CONTROL: ↑ fasting glucose; ↑ insulin; ↑ HbA1c, ↑ HOMA-IR, ↓ QUICKI. MARKERS OF INFLAMMATION: ↑ Amyloid A, ↑ NF-kB, ↑ IL-18. OXIDATIVE AND ANTIOXIDANT MARKERS: ↑ MDA, ↓ TAC. NEUROBEHAVIORAL TESTS: ↓ motor coordination by footprint assay; ↓ spatial working memory by Y-maze test; ↑ anxiety-like and exploratory behavior by open field test; ↓ spatial learning and retention memory by Morris water maze. HISTOPATHOLOGICAL: Frontal cerebral cortex tissue—disorganization of the layers, deformed neurons, the depletion of the cellular elements, inflammatory cell infiltration, and dilated congested blood vessels.
Bagheripour et al. [19]	- Female (weight = 170–180 g) and male Wistar rats (weight = 190–210 g); age 8 weeks. - Consumption of HFD (carbohydrate: 27.5%; protein: 14.5%; fat: 58%; lipid source: butter) for 91 days. - Administration of STZ (30 mg/kg i.p., single dose). - Confirmation of DM by fasting glycemia 7 days after STZ: DM if glycemia between 150 and 350 mg/dL. - Intervention for 56 days.	METABOLIC CONTROL: ↑ body weight; ↑ BMI; ↑ Lee index; ↑ abdominal circumference; ↑ thoracic circumference immediately and after 28 days, 56 days the intervention. WAT: ↑ Inguinal, ↑ Epididymal, ↑ Mesenteric, ↑ Retroperitoneal; BAT: ↓ Interscapular, ↓ Axillary; ↔ adiposity index. GLYCEMIC CONTROL: ↑ fasting glucose; ↑ area under the curve of serum glucose in the intraperitoneal (i.p.) glucose tolerance test; ↑ area under the curve of serum glucose in the i.p. pyruvate tolerance test immediately and after 28 days or 56 days of the intervention. LIPID PROFILE: ↑ TG, ↑ TC, ↑ LDL-c, ↑ HDL-c immediately and after 28 days or 56 days of the intervention. OXIDATIVE AND ANTIOXIDANT MARKERS: ↔ area under the curve of serum NOx and ↓ area under the curve of serum citrulline immediately and after 28 days or 56 days of the intervention.
Ghasemi et al. [20]	- Male Wistar rats weighing between 190 and 210 g and aged 8 weeks. - Consumption of HFD (carbohydrate: 27%; protein: 14,2%; fat: 58,8%; lipid source: sheep butter) for 14 days. - Administration of STZ (25 mg/kg i.p., single dose). - Confirmation of DM by fasting glycemia 7 days after STZ: DM if glycemia ≥ 150 mg/dL. - Intervention for 56 days.	METABOLIC CONTROL: ↑ body weight; ↓ food consumption, ↑ water intake. GLYCEMIC CONTROL: ↑ fasting glucose; ↑ insulin; ↓ Islets GIIS; ↑ HOMA 1-IR, ↑ HOMA2-IR, ↓ QUICKI. GENE EXPRESSION: mRNA expression in the pancreatic islets: NADPH oxidase (Nox) isoforms: ↑ Nox1, ↑ Nox2, ↔ Nox3, and ↑ Nox4; SOD isoforms: ↓ SOD1, ↓ SOD2, and ↔ SOD3; ↑ CAT; Gpx isoforms: ↓ GPX1 and ↓ GPX7; ↓ GR; TXN isoforms: ↓ TXN1 and ↔ TXN2; ↔ TXNRD1.
Tarighat-Esfanjani et al. [23]	- Male Wistar rats weighing above 220 g. - Consumption of HFD (carbohydrate: 48%; protein: 20%; fat: 32%; lipid source: rump oil) for 30 days. - Administration of STZ (35 mg/kg i.p., single dose). - Confirmation of DM by glycemia (not disclosed) 7 days after STZ: DM if glycemia > 250 mg/dL by oral glucose tolerance test (2 g/kg). - Intervention for 49 days.	METABOLIC CONTROL: ↓ body weight over the 49 days of the intervention. GLYCEMIC CONTROL: ↑ fasting glucose; ↔ insulin; ↑ HOMA-IR. LIPID PROFILE: ↔ TG; ↔ TC; ↔ VLDL; ↔ LDL-c. MINERAL’S LEVELS IN SERUM: No difference in Mg, Ca, Cu, P, and Zn.
Cai et al. [35]	- Male Wistar rats weighing between 250 and 280 g and aged 8 weeks. - Consumption of HFD (carbohydrate: 26.9%; protein: 15.6%; fat: 57.5%; lipid source: not disclosed) for 30 days. - Administration of STZ (35 mg/kg i.p., single dose). - Confirmation of DM by fasting glycemia (a not disclosed number of days after STZ): DM if glycemia > 200 mg/dL for two consecutive measurements. - Intervention for 56 days.	METABOLIC CONTROL: body weight lower over the 56 days of the intervention; ↑ food consumption, ↑ water intake. GLYCEMIC CONTROL: ↑ fasting glucose; ↑ insulin; ↑ HOMA-IR, ↓ HOMA-β, ↑ area under the serum glucose curve in the oral glucose tolerance test. LIPID PROFILE: ↑ TG, ↑ TC, ↑ LDL-c, ↓ HDL-c. CARDIOVASCULAR FUNCTION: ↔ LV end-diastolic dimension, ↑ ratio of E-wave and A-wave, ↔ isovolumic relaxation time, ↔ E-wave decline time, ↓ ejection fraction, ↓ fractional shortening, ↓ heart rate, ↔ cardiac output, ↑ BNP. HISTOPATHOLOGICAL: disordered cardiac tissues and ↑ lipid levels within the hearts, ↑ heart FFA and DAG contents. GENE AND PROTEIN EXPRESSION: - mRNA expression in cardiac tissue: ↑ PPARα, ↑ CD36, ↔ AMPK, ↑ FOXO1, and ↑ BNP. - Protein expression in cardiac tissue: ↑ PPARα, ↑ CD36, ↔ AMPK, and ↑ FOXO1.
A-Elgadir et al. [36]	- Male Wistar rats weighing between 200 and 210 g and aged 8–9 weeks. - Consumption of HFD (carbohydrate: 20%; protein: 20%; fat: 60%; lipid source: not disclosed) for 91 days. - Administration of STZ (45 mg/kg i.p., single dose). - Confirmation of DM by fasting glycemia 7 days after STZ: DM if glycemia ≥ 200 mg/dL - Intervention for 14 days, 56 days after being diagnosed with diabetes mellitus.	METABOLIC CONTROL: ↓ body weight. GLYCEMIC CONTROL: ↑ fasting glucose; ↑ insulin; ↑ HOMA-IR. LIPID PROFILE: ↑ TG, ↑ TC, ↑ LDL-c, ↓ HDL-c. CARDIOVASCULAR FUNCTION: ↓ heart rate, ↓ LV-developed pressure, ↓ contractility index, ↑ systolic blood pressure, ↑ CK-MB, ↑ Troponin C. HISTOPATHOLOGICAL: disarrayed muscle fibers with cytoplasmic vacuoles and noticeable apoptotic alterations. PROTEIN EXPRESSION: Protein expression in the rigth atrial tissue: ↑ JAK-2, ↑ STAT3, ↑ iNOS, ↑ Casp3.
Gharaat; Choobdari; Sheykhlouvand [37]	- Male Wistar rats weighing between 170 and 190 g and aged 10 weeks. - Consumption of HFD (carbohydrate: 35%; protein: 10%; fat: 55%; lipid source: animal fat oil) for 28 days. - Administration of STZ (40 mg/kg i.p., single dose). - Confirmation of DM by glycemia (not disclosed) 3 days after STZ: DM if glycemia > 300 mg/dL. - Intervention for 42 days.	METABOLIC CONTROL: ↑ body weight pre-intervention, ↔ at the end of 42-day intervention and after physical exercise, ↔ VO_max_ test pre-intervention, at the end of 42-day intervention, and after physical exercise. GLYCEMIC CONTROL: ↑ fasting glucose; ↔ insulin; ↑ HOMA-IR in pre-intervention, at the end of 42-day intervention, and after physical exercise. CARDIOVASCULAR FUNCTION: ↑ LV end-diastolic volume, ↑ LV end-systolic volume, ↓ ejection fraction. PROTEIN CONCENTRATION: ↑ Casp9, ↓ CAT in serum PROTEIN EXPRESSION: Protein expression in the left ventricle myocardium: ↑ p53.
Mahmoud et al. [38]	- Male Wistar rats weighing between 150 and 160 g and aged 6–7 weeks. - Consumption of HFD (carbohydrate: 17%; protein: 25%; fat: 57%; lipid source: not disclosed) for 42 days. - Administration of STZ (35 mg/kg i.p., single dose). - Confirmation of DM by glycemia 7 days after STZ: DM if fasting blood glucose level > 150 mg/dL and non-fasting blood glucose levels > 250 mg/dL. - Intervention for 14 days, 56 days after being diagnosed with diabetes mellitus.	GLYCEMIC CONTROL: ↑ fasting glucose; ↑ insulin; ↑ HOMA-IR. CARDIOVASCULAR FUNCTION: ↑ LV end-diastolic dimension, ↑ LV end-systolic dimension, ↓ ejection fraction, ↓ fractional shortening, ↔ E-wave (peak early diastolic filling velocity) end study, ↑ A-wave (late diastolic filling velocity) end study, ↓ E-wave/A-wave end study, ↑ DT end study. MARKERS OF INFLAMMATION: ↑ TNF-α, ↑ IL-1β, ↑ NRF2. HISTOPATHOLOGICAL: fiber degeneration, size variation, thinning, corrugation, tears, heterogeneous staining, preserved intercalated disks, dark nuclei, vacuolations, and small blood vessels, ↑ in % connective tissue area both interstitial and perivascular. PROTEIN EXPRESSION: Protein expression in cardiac tissue: ↑ Casp-1, ↑ iNOS.
Swain et al. [39]	- Male and female Wistar rats weighing between 150 and 180 g. - Consumption of HFD (carbohydrate: 17%; protein: 25%; fat: 58%; lipid source: not disclosed) for 15 days. - Administration of STZ (40 mg/kg body weight, i.p., single dose). - Confirmation of DM by fasting glycemia 7 days after STZ: DM if glycemia > 300 mg/dL. - Intervention for 28 days.	GLYCEMIC CONTROL: ↑ fasting glucose; ↓ insulin; ↑ HbA1c, ↑ HOMA-IR, ↓ HOMA-β, ↓ QUICKI. MARKERS OF INFLAMMATION: ↑ TNFα, ↑ NF-kB, ↑ hs-CRP in serum, liver, and pancreas. PROTEIN EXPRESSION: - Protein expression in the liver: ↑ GLUT2. - Protein expression in the skeletal muscle: ↓ GLUT4.
Khosravi et al. [40]	- Male Wistar rats weighing between 188 and 212 g and aged 8 weeks. - Consumption of HFD (carbohydrate: 20%; protein: 20%; fat: 60%; lipid source: not disclosed) for 56 days. - Administration of STZ (35 mg/kg i.p., single dose). - Confirmation of DM by fasting glycemia 3 days after STZ: DM if glycemia > 300 mg/dL. - Intervention for 14 days.	METABOLIC CONTROL: ↔ body weight before induction, ↑ body weight after induction and after intervention. GLYCEMIC CONTROL: ↔ fasting glucose before induction, ↑ fasting glucose after induction and after intervention; ↔ insulin before induction, ↑ insulin after induction and after intervention; ↔ HOMA-IR before induction, ↑ HOMA-IR after induction and after intervention. OXIDATIVE AND ANTIOXIDANT MARKERS: ↓ GPX-4, ↑ MDA. PROTEIN CONCENTRATION: ↑ lactate in serum. PROTEIN EXPRESSION: Protein expression in the hippocampus: ↓ BDNF, ↓ SIRT1, ↓ MCT2, ↓ NRF2, ↓ p62, ↑ Keap1, ↓ PINK1, ↓ Parkin, ↓ Amyloid Beta, ↓ hyperphosphorylated Tau protein.

Legend: DM—diabetes mellitus; HFD—high-fat diet; STZ—streptozotocin; i.p.—intraperitoneal; ↓ decrease; ↑ increase; ↔ no change. A-SMA—α-smooth muscle actin; aAKT2—RAC-beta serine/threonine-protein kinase; ACC—Acetyl CoA carboxylase; AGRP—Agouti-related protein; ALP—alkaline phosphatase; AKT—protein kinase b; ALT—Alanine aminotransferase; AST—Aspartate aminotransferase; BAT—brown adipose tissue; BDNF—brain-derived neurotrophic factor; BNP—B-type natriuretic peptide; BMI—body mass index; Casp3—Caspase-3 protein; Casp9—Caspase-9 protein; CART—amphetamine-related transcript; CAT—Catalase; FAS—fatty acid synthase; CK-MB—creatine kinase–MB; TC—total cholesterol; DGAT2—Diacylglycerol acyltransferase; DT—deceleration time; ERK1/2—extracellular signal-regulated kinase ½; FFA—free fatty acid; FOXO1—Forkhead box O1 protein; G6pase—glucose 6 phosphatase; GIIS—glucose-induced insulin secretion; GLUT4—glucose transporter type 4; GLP1—Glucagon-like peptide type 1; Gpx—Glutathione peroxidase; GR—glutathione reductase; GSH—L-glutathione; HbA1c—glycated hemoglobin; hs-CRP—high-sensitivity C-reactive protein; HDL-c—HDL cholesterol (high-density lipoprotein); HOMA-IR—homeostasis model assessment—insulin resistance; HOMA2-IR: updated HOMA-IR; HOMA-β—Homeostasis Model Assessment—β-cell function; HOMA-IS—homeostasis model assessment—insulin sensitivity index; IL-1β—Interleukin 1β; IL-6—Interleukin 6; IL-8—Interleukin 8; iNOS—inducible nitric oxide synthase; IR—insulin receptor; Irs1—insulin receptor substrate 1; ISI—insulin sensitivity index; JAK-2—Janus kinase 2; JNK—c-Jun—N-terminal kinase; Keap1—Kelch-like ECH-associated protein 1; LDH—lactate dehydrogenase; LDL-c—LDL cholesterol (low-density lipoprotein); LEP-R—leptin receptor; MDA—Malondialdehyde; MCT2—monocarboxylate transporter 2; miR-125a-5p—micro-RNA125a-5p; mRNA—messenger RNA; Mtor—mammalian target of rapamycin; NEFA—non-esterified fatty acid; NF-kB—nuclear factor kappa B; NOx—nitrogen oxide; NPY—neuropeptide Y; NRF2—nuclear factor erythroid 2-related factor 2; p-AKT—phosphorylated protein kinase B; p-AMPK—phosphorylated adenosine monophosphate-activated protein kinase; p-ERK1/2—phosphorylated extracellular signal-regulated kinase 1/2; p-FOXO1—phosphorylated forkhead box O1 protein; p-JAK-2—Janus kinase 2 phosphorylated; p-STAT3—phosphorylated signal transducer and activator of transcription 3; Parkin—RING domain-containing E3 ubiquitin ligase; PCK2—phosphoenolpyruvate carboxykinase; PEPCK—Phosphoenolpyruvate carboxykinase; PI3k—Phosphoinositide 3-kinase; PINK1—PTEN-induced kinase 1; PKR—protein kinase R; POMC—pro-opiomelanocortin cocaine; PPARγ—peroxisome proliferators-activated receptor gamma; QUICKI—quantitative insulin sensitivity check index; Raptor—regulatory-associated protein of MTOR; SCD1—Stearoyl-CoA desaturase-1; SIRT1—NAD-dependent sirtuin deacetylase-1; SREBP1c—sterol response element binding protein 1c; STAT3—signal transducer of targeting and activator of transcription 3; SOCS3—suppressor of cytokine signaling; SOD—superoxide dismutase; TAC—total antioxidant capacity; TG—triglycerides; TGF-β—transforming growth factor beta; TNFα—tumor necrosis factor; TXN—Thioredoxin; TXNRD1—thioredoxin reductase; VLDL—very low density lipoprotein; VO_max_—maximal oxygen consumption; WAT—white adipose tissue. * Comparisons between diabetic control and normal control.

**Table 2:** Risk of bias of included studies obtained by the SYRCLE tool.

Author/Year	Selection Bias			Performance Bias		Detection Bias		Friction Bias	Reporting Bias	Other Sources of Bias		
	Random Allocation of Groups	Similar Groups in the Baselines	Blind Group Allocation	Random Housing	Blind Interventions	Random Outcome Assessment	Blinded Outcome Assessment	Report of Withdrawals	Selective Outcome Reporting	DM2 Cut-Off Point	Blood Glucose Results to Confirm DM2	Blood Glucose Results During/After Intervention
Bem et al. [14]	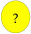	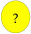	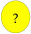	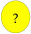	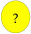	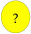	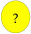	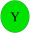	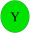	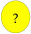	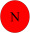	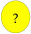
Gheibi et al. [18]	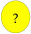	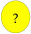	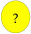	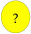	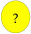	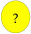	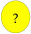	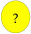	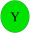	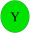	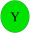	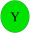
Sathiyabama et al. [25]	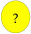	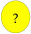	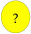	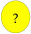	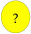	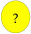	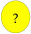	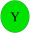	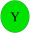	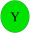	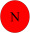	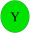
Sohrabipour et al. [26]	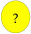	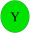	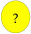	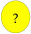	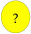	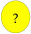	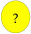	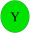	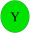	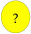	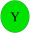	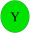
Abel-Hamid et al. [21]	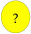	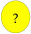	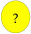	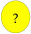	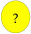	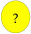	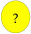	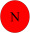	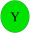	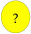	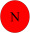	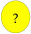
Ghiasi et al. [27]	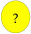	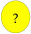	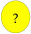	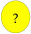	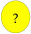	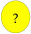	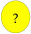	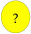	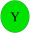	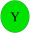	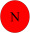	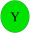
Omidi et al. [28]	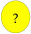	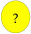	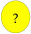	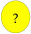	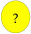	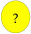	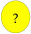	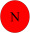	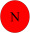	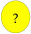	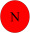	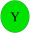
Xiang et al. [22]	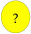	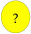	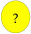	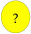	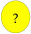	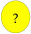	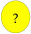	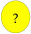	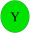	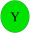	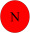	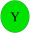
Zhang et al. [29]	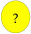	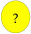	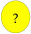	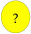	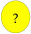	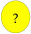	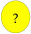	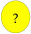	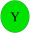	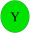	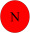	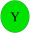
Lv et al. [30]	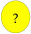	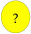	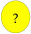	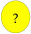	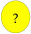	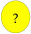	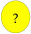	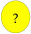	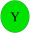	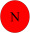	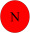	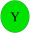
Mangali et al. [31]	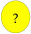	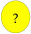	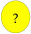	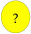	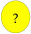	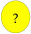	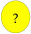	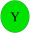	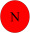	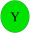	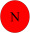	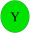
Xu et al. [32]	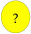	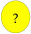	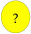	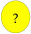	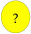	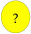	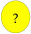	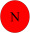	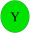	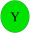	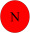	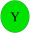
Kolefer; Miaffo; Ponka [33]	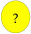	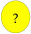	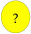	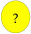	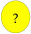	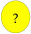	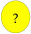	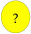	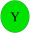	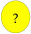	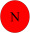	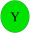
Rezazadeh et al. [24]	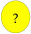	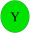	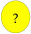	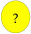	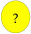	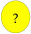	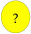	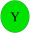	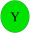	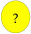	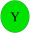	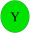
Zelinskaya et al., [34]	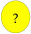	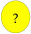	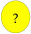	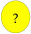	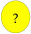	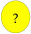	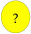	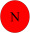	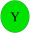	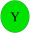	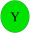	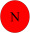
Khoramipour et al. [15]	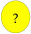	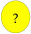	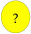	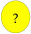	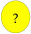	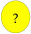	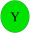	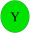	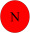	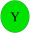	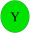	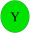
Vijay; Vellapandian [16]	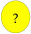	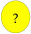	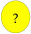	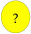	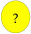	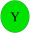	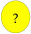	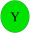	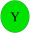	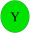	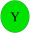	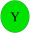
Salem et al. [17]	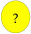	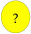	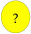	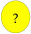	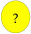	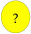	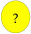	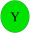	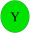	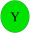	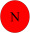	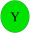
Bagheripour et al. [19]	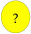	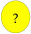	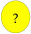	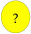	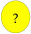	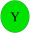	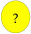	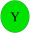	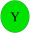	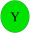	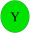	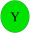
Ghasemi et al. [20]	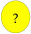	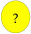	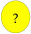	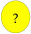	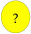	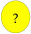	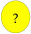	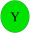	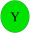	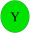	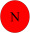	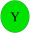
Tarighat-Esfanjani et al. [23]	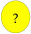	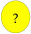	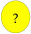	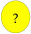	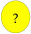	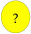	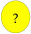	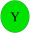	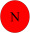	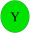	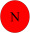	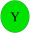
Cai et al. [35]	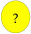	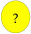	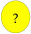	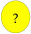	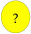	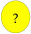	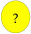	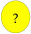	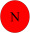	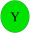	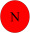	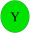
A-Elgadir et al. [36]	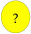	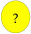	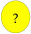	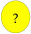	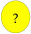	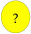	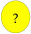	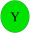	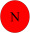	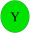	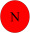	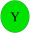
Gharaat; Choobdari; Sheykhlouvand [37]	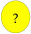	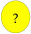	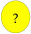	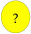	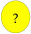	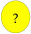	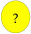	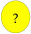	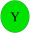	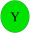	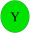	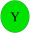
Mahmoud et al. [38]	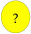	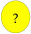	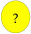	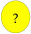	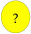	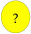	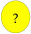	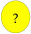	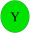	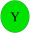	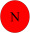	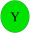
Swain et al. [39]	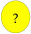	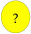	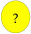	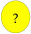	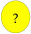	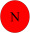	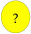	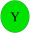	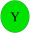	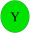	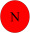	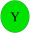
Khosravi et al. [40]	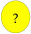	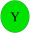	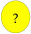	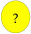	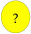	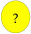	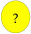	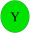	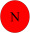	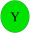	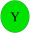	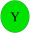

Key: Y (green) = yes; N (red) = no; ? (yellow) = unclear.

## 4. Discussion

All of the experimental models included in this review confirmed the induction of diabetes and reported increased insulin resistance, among other characteristic alterations, when STZ was administered intraperitoneally in a single dose ranging from 25 to 45 mg/kg of body weight, after a minimum period of maintaining the animals on HFD for 21 days. Almost all of the studies included in this review used male rats weighing between 150 and 280 g for DM2 induction protocols. In summary, the key outcomes include hyperglycemia, hyperinsulinemia, insulin resistance, dyslipidemia, increased inflammation and oxidative stress, and structural changes and changes to the expression of proteins related to insulin signaling and carbohydrate and lipid metabolism in various tissues.

The preference for male animals is based on studies showing that sex affects the variations observed during diabetes induction, with females being less susceptible to STZ [41]. Kang et al. [42] reported that xenoestrogens can reverse the reduction in insulin and the mRNA expression of insulin transcriptional regulators (Pdx1, Mafa, and Neurod1) of pancreatic islet beta cells induced by STZ, and this effect is related to the activation of NF-kB, which produces anti-apoptotic effects. This shows the protective role of estrogens against the action of STZ, supporting the idea that female rats are less sensitive to the action of STZ than males.

Female rats have also been used in research studies, although less frequently. Rezazadeh et al. [24] showed that male and female Wistar rats exhibited no discernible differences in body weight, glycemic and metabolic parameters, and insulin signaling pathways when induced with DM2 through the consumption of a high-fat diet (composed of 17% carbohydrates, 58% lipids, and 25% proteins) for 84 days coupled with STZ administration (a single dose of 35 mg/kg, i.p.). Similarly, Bagheripour et al. [19] suggested the successful induction of T2DM in male and female rats after the consumption of an HFD containing 58% fat for 91 days and the administration of STZ (a single dose of 30 mg/kg, i.p.). It is important to note that the prolonged duration of diet consumption, paired with the high lipid content and additional intervention period, potentially contributed to the similar outcomes witnessed for male and female rats, enabling the necessary metabolic changes for induction to occur. Animal weight and age are crucial factors that can influence the induction of diabetes. In this review, most of the studies included used rats that were young (4 to 16 weeks) and had a lower weight (150–280 g). Only one study used male Wistar rats weighing more than 250 g and aged between 20 and 24 weeks. This particular study revealed that glycemic dysregulation and no changes in lipid profile were observed in animals at the end of 56 days of post-induction intervention with an HFD (48% carbohydrates, 32% lipids, and 20% proteins) for 30 days coupled with STZ administration (35 mg/kg, i.p., single dose) [28].

In contrast, Xu et al. [32] discovered that male Wistar rats weighing between 180 and 220 g fed a high-fat diet (41% carbohydrates, 24% lipids, and 24% proteins) for 28 days, coupled with STZ administration (30 mg/kg, i.p., single dose) and monitored for 56 days, not only experienced changes in glycemic control but also dysregulations of lipid profile, liver function, and the gene expression of pathways related to gluconeogenesis. Although the diet’s lipid source is not clear, the animals in Xu’s study [32] were lighter and presented more severe metabolic dysfunctions compared to Omidi’s study [28], even when receiving a diet with a lower percentage of lipids and a lower dose of STZ, which suggests that young, lighter weight rats have a lower resistance to the induction of DM2.

In a study evaluating the impact of body weight on susceptibility to the induction of type 1 diabetes (DM1) by STZ in male Wistar–Glaxo rats, younger and light-weight animals were found to be less sensitive to the action of STZ; thus, higher doses of STZ are required to produce effects comparable to older and heavy-weight animals [43]. Moreover, Cheng et al. [44] found that post-weaning male Sprague Dawley rats (3 weeks old) fed an HFD for 8 weeks demonstrated greater and faster weight gain and increased blood glucose and white adipose tissue when compared to adult rats (8 weeks old); this implies younger rats are more susceptible to the induction of metabolic dysfunction.

Based on observations from previous studies [43,44], it appears that an HFD and STZ have differing effects depending on the age and weight of the animals. Specifically, an HFD was found to be more potent in younger and light-weight animals, while STZ had a greater impact on older and higher weight animals. Additionally, the more severe metabolic changes in the animals in the study by Xu et al. [32] that had a lower weight indicate that an HDF has a greater impact than STZ administration in terms of inducing DM2.

Consuming high-fat diets can cause rats to take in more calories than they normally would, leading to obesity and other weight-related issues. Over time, this type of diet can result in a range of changes within the body, including increased weight, lipid buildup, hyperglycemia, hyperinsulinemia, insulin resistance, inflammation, and damage to organs such as the liver, adipose tissue, and pancreas [45].

High-fat diets are those that contain a minimum of a 10% increase in fat content compared to a standard diet, with carbohydrates being replaced to increase calorie intake. These diets have been shown to have a significant impact on the liver, skeletal muscle, and adipose tissue [46]. A fat content of 10–12% is used as a baseline for comparison [47]. However, variations in the type and concentration of fats, as well as the duration of the diet, can result in different pathological characteristics, despite all studies in this review meeting the criteria for a high-fat diet.

Using the HFD + STZ model in male Wistar rats (190–210 g), Gheibi et al. [18] demonstrated that after 14 days of diet (27.5% carbohydrate, 58% lipid, and 14.7% protein) administration and before STZ application, the animals showed an increase in body weight, food and water consumption, hyperglycemia, hyperinsulinemia, and an increase in serum lipid profile markers. On the other hand, Magalhães et al. [8] found no differences in food and water consumption, diuresis, body weight, and serum glycemia in male Wistar rats (240–250 g) subjected to diabetes induction with standard feed enriched with sugar and lard (51% carbohydrates, 35% lipids, and 14% proteins) for 12 days prior to the application of the STZ.

The different outcomes described in this review seem to be connected to variations in fat concentrations and the types of lipid sources in the diets. Diets with a high percentage of fat are associated with more severe metabolic alterations that include hyperglycemia, hyperinsulinemia, and increased insulin resistance. These alterations are often accompanied by an escalation in oxidative stress, inflammation, and lipid buildup in the liver, adipose, and pancreatic tissues [45].

In the HFD + STZ model, Tarighat-Esfanjani et al. [23] observed an increase in fasting blood glucose and HOMA-IR, but without changes in insulin and lipid profile after the consumption of an HFD for 30 days (containing 32% lipids of animal origin), the administration of 35 mg/kg of STZ i.p. in a single dose, and a 49-day intervention period. Cai et al. [35], in turn, identified an increase in the fasting blood glucose, insulin, and HOMA-IR associated with changes in the lipid profile after the consumption of an HFD containing 57% fat for 30 days, with the administration of 35 mg/kg of STZ i.p. in a single dose, and a 56-day intervention period. Similarly, other authors also observed more intense metabolic changes with the use of diets containing high percentages of fat. In terms of lipid sources, rats fed diets rich in saturated fatty acids have a higher body weight, increased body fat, and greater insulin resistance [48]. In this review, the most cited fat source is lard [14,22,24], but other sources of saturated fatty acids [18] and associations between two different lipid sources [21] have also been described, although most authors do not clarify the source included in their studies.

The prolonged consumption of a high-fat diet (HFD) can contribute to the development of pathological changes in DM2. A study by Rodríguez-Correa et al. [46] found that consuming an HFD for an extended period is linked to weight gain, visceral fat accumulation, and elevated serum triglyceride levels. In this review, however, an HFD was used before the injection of STZ, and the duration of consumption varied widely from 14 to 84 days.

A study evaluating the effect of the prior consumption of HFD for 3, 4, and 5 weeks (21, 28, and 35 days) and the injection of STZ (25 mg/kg, i.p., single dose) after one week revealed that Wistar rats of both sexes (200–300 g) demonstrated an increase in fasting glycemia and dyslipidemia, with no changes in body weight or serum insulin levels in the group fed an HFD for 3 weeks. However, only the groups fed an HFD for 4 and 5 weeks showed a reduction in insulin sensitivity and higher glycemic values. The HFD used in the study contained lard and cholesterol as sources of lipids, but the percentage of lipids used is not described in the study [49].

In this review, eight of the studies used HFD consumption times shorter than 28 days [14,16,17,18,20,25,34,39]. Of these, six studies used an HFD between 14 and 21 days; however, they did not provide any data on insulin sensitivity when confirming diabetes, with results reported after the end of the intervention (between 21 and 69 days), a period in which HFD consumption is maintained.

In studies conducted by Gheibi et al. [18] and Sathiyabama et al. [25], male Wistar rats of comparable weight were subjected to a high-fat diet (HFD) for 14 days, showing, hyperglycemia, greater insulin resistance, and dyslipidemia occurring after 5 and 7 days after STZ injection, respectively. However, Sathiyabama et al. [25] used a lower lipid percentage (31% carbohydrates, 55% lipids, and 14% proteins) and a higher dose of STZ (40 mg/kg, i.p., single dose) compared to Gheibi et al. [18] who used an HFD (27.5% carbohydrates, 58% lipids, and 14.7% proteins) and STZ (25 mg/kg, i.p., single dose) to induce these changes. Despite the differences between the studies, the similar findings suggest that HFD consumption within 19 to 21 days (approximately 3 weeks) is able to promote glycemic and lipid changes that vary according to the percentage of lipids in the diet and the dose of STZ administered.

STZ is a diabetogenic drug that contains deoxyglucose molecules in its chemical structure, giving it selectivity for GLUT 2 receptors in pancreatic beta cells. When it binds to these cells, it has cytotoxic effects mediated by DNA methylation mechanisms, nitric oxide production, and the generation of reactive oxygen species [50]. In experimental studies with rats, STZ doses can be classified as high (above 65 mg/kg), moderate (between 55 and 40 mg/kg), or low (less than 35 mg/kg) [18,51], and their increase has been associated with a gradual reduction in pancreatic insulin content [52].

Omolaoye et al. [53] studied the isolated effects of different doses of STZ (30 and 60 mg/kg body weight) on the male reproductive function of adult Wistar rats (240–290 g). In this study, there was a reduction in the body weight of the diabetic animals and an increase in water intake, food consumption, and glycemia only in the group that received STZ at a dose of 60 mg/kg. In turn, the STZ at the dose of 30 mg/kg showed no significant difference in relation to food consumption, water intake, and blood glucose [53]. This implies that a 30 mg/kg dose of STZ alone is not effective at inducing diabetes.

Although it does not affect glycemia, a single administration of lower doses of STZ causes functional defects in beta cells. Rossi and Heldstab [54] examined the effects of latent diabetes induced by streptozotocin on the pancreas of rats and observed a reduction in the area of the pancreatic islets as well as a lower amount of insulin in the pancreatic islets of normal animals, but higher in symptomatic diabetics. This demonstrates the role of STZ in producing mild impairment of pancreatic beta cells when administered at low doses.

The use of low doses of STZ changes the metabolic profile of Wistar rats fed HFD. Gheibi et al. [18] showed that, at the end of the 14-day HFD, Wistar rats displayed an increase in body weight, food consumption and water intake, hyperglycemia, hyperinsulinemia coupled with an increase in triglycerides, total cholesterol, and its lipoprotein fractions. Meanwhile, 7 days after STZ administration, animals exhibited a reduction in body weight, food consumption, and water intake, with a drastic increase in glycemia and a return of serum insulin concentration to levels similar to the control. Additionally, the serum levels of triglycerides, total cholesterol, and LDL cholesterol increased, while HDL cholesterol levels decreased.

The reduction in insulin, weight, and food and water consumption after the application of the STZ may be due to its acute toxicity against beta cells, since the authors prove in the study that these parameters began to increase during the intervention period. Goyal et al. [41] explain that the action of STZ in terms of inducing diabetes has three phases: the first phase occurs two hours after its administration, during which hepatic glycogen is broken down to produce transient hyperglycemia and hypoinsulinemia; the second phase begins 10 h later, with hypoglycemia mediated by massive insulin release from ruptured beta cells; and the third begins 24 h later, when stable hyperglycemia and hypoinsulinemia are established. Therefore, in the HFD + STZ model, STZ seems to boost the effect of the diet, which is an essential step towards the development of diabetes mellitus itself.

Wickramasinghe, Attanayake, and Kalansuriya [11] confirmed the role of STZ in producing different phenotypes of the HFD + STZ model by comparing the effects of HFD feeding only, (54.6% carbohydrates, 24.8% lipids, and 12.8% proteins) for 28 days, and after the administration of three different doses of STZ (30, 40, and 50 mg/kg, i.p., single dose) in male Wistar rats weighing between 135 and 165 g. The authors’ findings indicate that the animals fed with an HDF only showed a profile similar to the pre-diabetes stage with increased body weight and insulin resistance, changes in serum lipids, but not hyperglycemia. On the other hand, the administration of doses of 30 and 40 mg/kg after the consumption of the HFD produced a close phenotypic resemblance to DM2 with increased body weight, hyperglycemia, hyperisulinemia, and dyslipidemia. However, STZ at the 50 mg/kg dose induced a condition similar to advanced-stage DM, with a reduced body weight and severe glycemic and lipid alterations. The differences between DM1 and DM2 at an advanced stage can be subtle, since DM2 displays similar characteristics to DM1 as the disease progresses. Srinivasan et al. [55] observed hyperglycemia, hypoinsulinemia, and reduced body weight in animals submitted to diabetes induction using an HFD for 2 weeks and moderate doses of STZ (45 and 55 mg/kg) compared to normal animals. However, treatment with insulinotropic agents and insulin sensitizers was not effective at abolishing the increase in blood glucose, indicating that these animals had characteristics similar to type 1 diabetes.

In this review, only one study reproduced the HFD + STZ model using a dose above 40 mg/kg [52]. The author reported an increase in blood glucose, insulin, HOMA-IR, and changes in the lipid profile compatible with dyslipidemia after induction using an HFD with 60% fat for 91 days and the administration of 45 mg/kg of STZ i.p. in a single dose. These findings indicate that a high dose of STZ is not the main trigger for the induction of type 2 diabetes, but rather the fat percentage and the duration of diet supply. Higher doses of STZ, even after HFD consumption, may not adequately reproduce the pathological features of T2DM if the fat percentage and the duration of diet provision are not sufficient to produce metabolic changes.

Akinlade, Owoyele, and Soladoye [56] described the expected differences between DM1 and DM2 by comparing models of DM1 (STZ 55 mg/kg in a single dose) and DM2 (HFD with 45% lipids for 28 days and STZ 25 mg/kg, single dose). Their evidence showed a progressive loss of body weight with no relevant changes in serum lipids and insulin resistance in DM1, whereas there was an increase in body weight and marked changes in serum lipids and insulin resistance in DM2. Differences in the glucose tolerance curves were also observed—it reached its peak in the first 30 min and declined more rapidly in DM1 compared to DM2, it attained its maximum value at 60 min, and declined slightly in the following periods. Pathological changes in the pancreas are more significant in animals with DM1.

In humans, the progression from pre-diabetes to diabetes requires, in addition to glycemic changes and changes in insulin function, a significant reduction in the mass of functional beta cells. In a prospective cohort study involving 6538 British people without diabetes at the start of the study, individuals who developed diabetes showed a rapid rise in fasting and post-load blood glucose levels 3 to 6 years before diagnosis and a marked reduction in insulin sensitivity 5 years before diagnosis. Pancreatic beta-cell function increased 3 to 4 years before diagnosis, and then declined until diabetes was diagnosed [57].

Insulin resistance in patients with type 2 diabetes is mediated by inflammatory and oxidative mechanisms, which in turn is driven by hyperglycemia and excess free fatty acids [58]. Reduced insulin production is also described in these patients due to the accumulation of lipid droplets in beta cells [59] and the overload suffered by beta cells when trying to compensate for insulin resistance with hyperinsulinemia [60]. In the HFD + STZ model, the metabolic changes produced by an HFD promote inflammatory and oxidative modifications that are intensified by a low dose of STZ, leading to the onset of type 2 diabetes.

To confirm the effectiveness of DM2 induction, blood glucose levels are measured to detect the presence of hyperglycemia. In the studies included in this review, the ways of assessing the effectiveness of the induction of diabetes vary in terms of the type of blood glucose levels used, the cut-off points, and the period after STZ administration. In general, the authors considered fasting glucose values above the range of 140 and 250 mg/dL and random glucose values above 300 mg/dL to classify the animals as diabetic. There is no standardization in the literature on the glycemia cut-off points to determine DM2 in animal models; however, it is recommended to consider animals that present glucose values greater than 150 mg/kg in the fasting state and above 200 mg/kg in the fed state [18,61].

After the confirmation of diabetes, the maintenance of an HFD is considered important for the maintenance of the experimental model. In a study by Guo et al. [62] that evaluated the stability of the type 2 diabetes model in Wistar rats induced by a high-fat diet for 8 weeks and combined with a single injection of 25 or 35 mg/kg of streptozotocin, it was shown that the reintroduction of a normal diet to diabetic rats for 4 weeks produced a reversal of the characteristics of diabetes. Thus, the maintenance of the HFD throughout the experimental period is necessary for the stability of the model.

Induction with STZ + HFD produces a variety of other biological effects that have been described after the intervention period. Concerning changes in glycemic control, increased glycemia, glycated hemoglobin [14,17,18,22,24,30,32,39], and insulin resistance were reported (by all studies included in the results). Serum insulin showed variations across studies, demonstrating an increase in almost all included studies—four studies noticed a reduction [15,16,21,26], one study observed no changes [23] and three others did not measure this parameter [19,24,31]. Increased blood glucose levels in glucose tolerance tests and insulin tolerance tests were identified in almost half of the studies analyzed [16,18,19,22,25,26,30,32,35].

Metabolic control information that included food intake and body weight varied across studies. Most authors [15,16,18,19,20,21,25,33,37] observed an increase in body weight, while three authors [14,31,40] observed no differences between the normal and diabetic groups, and the other half of the studies reported reductions in body weight. Food consumption was increased in six of the studies [18,21,25,30,33,35], while two showed no difference in this parameter [22,31] and three other studies showed a reduction in food intake [15,20,26]—this variation in results seems to be connected to the methodological differences in each study.

Regarding the biochemical markers of the lipid profile, almost all authors described an increase in triglycerides and cholesterol, except for Tarighat-Esfanjani et al. [23] and Omidi et al. [28] who observed no significant differences in either parameter, and Xiang et al. [22], who showed no differences in total cholesterol. Most studies reported reduced HDL cholesterol, only one study observed an increase in this parameter [18], and three other studies showed no differences in terms of this parameter [23,28,30]. On the other hand, an increase in the LDL-c levels was observed in most of the included studies. Additionally, some studies revealed increases in liver function markers (AST and ALT) [16,22,25,32,33] and renal function markers (urea and creatinine) [16,25,33].

Further hormonal changes have been described, such as increased leptin [14,15,29], increased pro-inflammatory cytokines IL-1β, IL-6, IL-8, and TNFα [14,17,18,21,30,31,38,39], and decreased adiponectin [23]. Moreover, some studies reported changes in oxidative stress markers, which includes increases in lipid peroxidation, the generation of reactive oxygen species, and reduced antioxidant enzyme activity [16,18,19,27,31,33].

In relation to tissue alterations, studies have highlighted a reduction in the content and secretion of insulin in the pancreas, a disorder in the architecture of the pancreatic islets, and a reduction in the density of beta cells [16,18,25,27]. The adipose tissue showed swollen cells and a reduction in adiponectin [25]. The liver developed hepatic steatosis with lobular inflammation, increased triglycerides, and decreased hepatic glycogen [16,21,29,30]. In addition, an increase in heart weight and fibrosis area, increased cardiac markers [35,36,38], and elevated heart rate and blood pressure [31], as well as changes in vascular contractility, have been reported in the studies [14,34].

**Figure 2:**
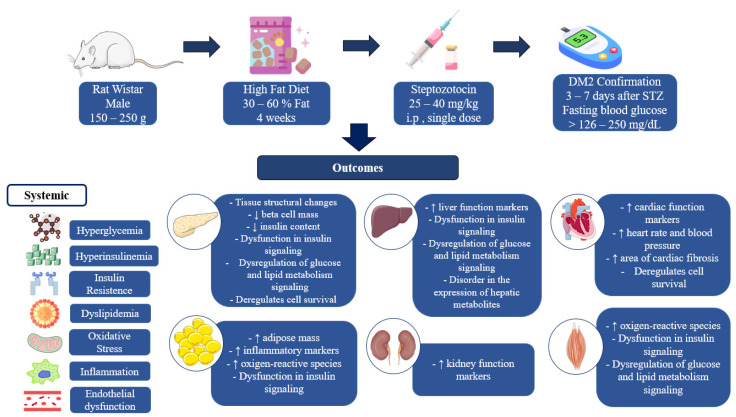
Method of induction of type 2 diabetes using HFD + STZ and its main outcomes.

The dysregulation of the insulin signaling pathway was identified in adipose tissue, liver, and skeletal muscle [14,18,22,25], and the dysregulation of glucose and lipid metabolism signaling was observed in the pancreas, liver, and skeletal muscle [22,25,26,29,32]. There was increased expression of NF-kB in visceral fat [21] and iNOS in muscle and adipose tissue [18]. Alterations in the transcriptional control of regulatory pathways, apoptosis, and cell proliferation were pointed out in the included studies [27,31]. Figure 2 summarizes the main characteristics of DM2 induction using the HFD + STZ model and its main pathological outcomes.

Some limitations were observed that made it impossible to conduct a more detailed analysis. There was a great variability between the studies due to the diversity of the authors’ objectives. Additionally, the absence of detailed information on the source of the lipids used in the HFD and the type of glycemia assessed in some of the studies made it challenging to compare the data and identify the causes of the outcomes more precisely. Additionally, the risks of bias identified may contribute to inaccuracies in the analysis.

## 5. Conclusions

The results of this investigation indicate that the use of young male rats fed diets containing high percentages of fat for at least 3 weeks followed by low doses of STZ are effective at producing metabolic, histological, inflammatory, oxidative, and metabolic signaling pathway changes in different tissues that are compatible with type 2 diabetes. Furthermore, although the model requires the association of STZ and an HFD, the latter has a greater impact on inducing disease characteristics. Thus, the HFD + STZ model reproduces not only the pathological characteristics but also the mechanisms of development of DM2 in humans.

## Data Availability

Data will be made available upon request.
